# European Headache Federation recommendations for placebo and nocebo terminology

**DOI:** 10.1186/s10194-020-01178-3

**Published:** 2020-09-25

**Authors:** Dimos D. Mitsikostas, Charlotte Blease, Elisa Carlino, Luana Colloca, Andrew L. Geers, Jeremy Howick, Andrea W. M. Evers, Magne A. Flaten, John M. Kelley, Irving Kirsch, Regine Klinger, Antoinette MaassenVanDenBrink, Daniel E. Moerman, Petros P. Sfikakis, Lene Vase, Tor D. Wager, Fabrizio Benedetti, on behalf of the European Headache Federation

**Affiliations:** 1 1st Neurology Department, Aeginiton Hospital, Medical School, National and Kapodistrian University of Athens, 72-72 Vas. Sofia’s Avenue, 11528 Athens, Greece; 2 General Medicine and Primary Care, Beth Israel Deaconess Medical Center, Boston, MA USA; 3 Physiology and Neuroscience, University of Turin Medical School, Turin, Italy; 4 Departments of Pain Translational Symptoms Science and Anaesthesiology, School of Nursing and Medicine, University of Maryland Baltimore, Baltimore, MD USA; 5 Department of Psychology, University of Toledo, Toledo, OH USA; 6 Faculty of Philosophy, University of Oxford, Oxford, UK; 7 Health, Medical and Neuropsychology Unit, Institute of Psychology, Leiden University, Leiden, The Netherlands; 8 Department of Psychology, Norwegian University of Science and Technology, Trondheim, Norway; 9 Beth Israel Deaconess Medical Center, Program in Placebo Studies, Boston, MA USA; 10 Department of Anesthesiology University Medical Center Hamburg-Eppendorf, Center for Anesthesiology and Intensive Care Medicine, Hamburg, Germany; 11 Division of Pharmacology, Department of Internal Medicine, Erasmus MC, Rotterdam, The Netherlands; 12 University of Michigan-Dearborn, Dearborn, Michigan USA; 13 1st Department of Propedeutic and Internal Medicine, National and Kapodistrian University Medical School, Athens, Greece; 14 Department of Psychology and Behavioural Sciences, Aarhus University, Aarhus, Denmark; 15 Department of Psychological and Brain Sciences, Dartmouth College, Hanover, NH USA

**Keywords:** Placebo, Placebo response, Placebo effect, Nocebo, Nocebo response, Nocebo effect

## Abstract

**Background and aim:**

Despite recent publications, practitioners remain unfamiliar with the current terminology related to the placebo and nocebo phenomena observed in clinical trials and practice, nor with the factors that modulate them. To cover the gap, the European Headache Federation appointed a panel of experts to clarify the terms associated with the use of placebo in clinical trials.

**Methods:**

The working group identified relevant questions and agreed upon recommendations. Because no data were required to answer the questions, the GRADE approach was not applicable, and thus only expert opinion was provided according to an amended Delphi method. The initial 12 topics for discussion were revised in the opinion of the majority of the panelists, and after a total of 6 rounds of negotiations, the final agreement is presented.

**Results/recommendations:**

Two primary and mechanism-based recommendations are provided for the results of clinical trials: [1] to distinguish the *placebo* or *nocebo response* from the *placebo* or *nocebo effect*; and [2] for any favorable outcome observed after placebo administration, the term “*placebo response*” should be used, and for any unfavorable outcome recorded after placebo administration, the term “*nocebo response*” should be used (12 out of 17 panelists agreed, 70.6% agreement). The placebo or nocebo responses are attributed to a set of factors including those that are related to the medical condition (e.g. natural history, random comorbidities, etc.), along with idiosyncratic ones, in which the placebo or nocebo effects are attributed to idiosyncratic, or nonspecific mechanisms, exclusively (e.g. expectation, conditioning, observational learning etc.). To help investigators and practitioners, the panel summarized a list of environmental factors and idiosyncratic dynamics modulating placebo and nocebo effects. Some of them are modifiable, and investigators or physicians need to know about them in order to modify these factors appropriately to improve treatment. One secondary recommendation addresses the use of the terms “*placebo*” and “*nocebo*” (“*placebos*” and “*nocebos*” in plural), which refer to the triggers of the placebo/nocebo effects or responses, respectively, and which are inert agents or interventions that should not be confused with the placebo/nocebo responses or effects themselves (all panelists agreed, 100% agreement).

**Conclusion:**

The working group recommends distinguishing the term response from effect to describe health changes from before to after placebo application and to distinguish the terms placebo(s) or nocebo(s) from the health consequences that they cause (placebo/nocebo responses or effects).

## Introduction

The terminology surrounding placebo and nocebo research is not yet familiar to practitioners and trialists. In addition, researchers in the field are using the related terms in a different way. Besides the recent consensus on the clinical implications of placebo and nocebo effects for clinical practice [1], there are still unanswered questions related to placebo and nocebo response and effect, as well as to the exact meaning of the term “*placebo*” and “*nocebo*”. For example, it is not yet clear whether the adverse events (AEs) recorded in the placebo-treated arm of a clinical trial, should be called nocebo response or nocebo effect. Others introduced the term “drucebo” (a combination of DRUg and plaCEBO or noCEBO) to relate to a favorable or unfavorable outcome which results from the patients’ expectation exclusively and is not pharmacologically caused by the medication [2]. Introducing new terms may generate more confusion, however. To address this issue, the European Headache Federation appointed a panel of experts by recruiting researchers in the field of placebo, clinicians and pharmacologists to further clarify the terms related to the placebo administration in clinical science.

## Methods

Twenty investigators were approached and 18 agreed to participate in the working group (90% participation rate). The composition of the task force was based on the following criteria: researchers focused on placebo or nocebo research (17 approached, two declined to participate), clinicians with relevant work (two members, DDM and PPS) and a member of the Executive Board of the EHF (AMB). Because no data were required to answer the questions, the Grading of Recommendations Assessment, Development and Evaluation (GRADE) approach was not applicable, and thus only expert opinion was provided. The agreement process was performed according to the Delphi method [3]. The co-chairs (DDM and FB) arranged 12 relevant topics for discussion (Table 1) shared by email with the members of the working group. In each email round, panelists were instructed not to discuss among themselves and to send their feedback only to the facilitator (DDM). The facilitator collected all the answers and issued for each round a report with comments together with the co-chairman (FB). Six consecutive drafts of the recommendation manuscript were prepared and reviewed by all authors. Based on their comments and suggestions drafts 1 and 5 were edited (rounds 2 and 6). The final manuscript (round 7) drafted by the chairs of the panel (DDM and FB) and was reviewed by all members of the panel for final approval.

**Table 1:** The 12 initial topics for discussion with the relevant decisions and proportion of agreement

1. Definition of placebo *(placebos,* plural*).* All panelists agreed to include this topic, and all agreed to the recommended definition.	
2. Definition of placebo phenomenon. 15 out of 17 panelists agreed to withdraw this term in order to avoid further confusion.	
3. Definition of nocebo *(nocebos,* plural*)*. All panelists agreed to include this topic, and all agreed to the recommended definition.	
4. Definition of nocebo phenomenon. 15 out of 17 panelists agreed to withdraw this term in order to avoid further confusion.	
5. Should we distinguish the placebo or nocebo response from the placebo or nocebo effect and why? All panelists agreed to include this topic; 12 out of 17 panelists agreed to distinguishing the two terms.	
6. Placebo response definition. All panelists agreed to include this topic, and all agreed to the recommended definition.	
7. Placebo effect definition. All panelists agreed to include this topic, and all agreed to the recommended definition.	
8. Nocebo response definition. All panelists agreed to include this topic, and all agreed to the recommended definition.	
9. Nocebo effect definition. All panelists agreed to include this topic, and all agreed to the recommended definition.	
10. Should we use the term placebos or nocebos to include both placebo/nocebo responses and effects? All panelists agreed to include this topic, and all agreed to distinguishing the two terms.	
11. Are placebo and nocebo phenomena cognitive-like functions? 15 out of 17 panelists agreed to withdraw this topic because it was not fitted to the aim of the working group.	
12. Are there animal and human models for placebo and nocebo? 15 out of 17 panelists agreed to withdraw this topic because it was not fitted to the aim of the working group.	

## RESULTS and RECOMMENDATIONS

The 12 initial topics for discussion with the relevant decisions and proportion of agreement are presented in Table 1. All panelists agreed with the final recommendation, except for distinguishing the terms placebo/nocebo response from placebo/nocebo effect (70.5% agreement). One panelist left the panel due to disagreement over the definition of placebo response and placebo effect. The working group agreed upon the following definitions.

### Placebo (placebos, plural)

Substances and interventions are considered placebos when they lead to a beneficial outcome after administration or application, although their active ingredients lack this potential. Active ingredients include pharmacologically active compounds, properties, psychological interventions, physical manipulations and other (e.g. sham surgery, sham stimulation, etc.).

#### Comments

1. Placebo is a substance or an intervention and should be distinguished from the placebo response that includes any health change from before to after the placebo administration or application, as well as the placebo effect which includes health changes related to the placebo mechanisms exclusively (see below).
2. A placebo is not only the inert treatment, or the intervention per se, but also any signal from the surrounding context that accompanies the therapeutic ritual/act. Stimuli from the environment that have been shown to modify the consequences of the placebo administration and/or application are listed in appendix.
3. Placebos (or placebo agents/interventions) are administered in research and clinical settings either to create a positive expectancy and subsequent health improvement or to validate a new treatment/intervention in clinical trials.
4. Any health change occurring in a placebo treated group of participants in the context of a clinical trial, also occurs in the active treatment group, in addition to the potential effect of the active ingredient. Therefore, placebos should always be indistinguishable by both the investigator and the trial participant in clinical trials (notwithstanding that this aim may sometimes not be achieved).
5. A scientifically supported treatment could be a placebo when given for an unrelated condition (e.g. an antibiotic prescribed for a viral illness), thus placebos are relative to a particular patient and a particular disorder.
6. Any substance or intervention that is offered to a patient or a volunteer by informing him/her that a potential beneficial treatment is being delivered serves as placebo as well, in addition to any other biological effect related to the pharmacological properties of the substance or the physical manipulations. This explains why placebos are present in clinical practice, when a physician recommends a treatment.
7. Positive information from media may also serve as a placebo.

### Placebo response

The placebo response refers to any beneficial consequence of a therapeutic act (e.g., differences in symptoms from before to after treatment), which is made up of any cue in the surrounding context, and informing the patient or the volunteer that a potential beneficial treatment is being delivered. The placebo response consists of any favorable health change occurring from before to after a placebo administration or application (i.e., differences in symptoms from before to after treatment), thus including the natural history of the medical condition investigated, the regression of the medical condition to the mean along with those changes that are attributed to the placebo mechanisms exclusively (see below).

#### Comments

1. Placebo responses are recorded in research and clinical settings to either investigate the phenomenon or to validate a new treatment/intervention.
2. In clinical trials the beneficial outcomes seen after administration of a placebo, should be called placebo response, which also includes the placebo effect.

### Placebo effect

A placebo effect refers to those particular beneficial health changes that are observed after a placebo administration or application, which are attributed to the placebo mechanisms exclusively, e.g. expectation, conditioning, observational learning.

#### Comments

1. The placebo effect is better studied in human studies, although animal studies may help elucidate some of the mechanisms, for example conditioning, particularly pharmacological conditioning.
2. The placebo effect is calculated as the difference between a placebo or nocebo treated group/condition and a no-treated group/condition as this is the basis for deriving mechanisms.

### Nocebo (nocebos, plural)

Substances and interventions are considering nocebos when they lead to a negative outcomes after administration or application, although their active ingredients lack this potential. Active ingredients include pharmacological properties, psychological effects or physical manipulations.

### Comments

1. Nocebos are purposefully applied only for research to create a negative expectancy and outcome in order to investigate the origin of nocebo phenomenon. However, nocebos can also be found in clinical research and routine medical practice. For example, the safety information delivered by the investigators to the participants of a clinical trial or the AEs listed in the leaflet of drug packages, may induce negative expectations serving as nocebos.
2. Like placebo, nocebo is characterized by the surrounding context that consists of any stimulus or information from the environment (appendix).

### Nocebo response

The nocebo response refers to any unfavorable consequence of a therapeutic act (e.g., differences in symptoms from before to after treatment), which is made up of any cue in the surrounding context, and informing the patient or the volunteer that a negative outcome may occur. Thus, nocebo response includes the natural history of the medical condition in question, spontaneous worsening of the symptoms, as well as random comorbidities, among several other components that are attributed to the nocebo effect.

#### Comments

1. To investigate or observe a nocebo response the administration of a nocebo is required. A nocebo response may occur unintentionally as well.
2. Assessing the AEs, or any unfavorable outcome, in patients who are treated with placebo, monitors the nocebo response in clinical trials.
3. Nocebo response is associated with lower adherence to the therapeutic intervention and higher rates of treatment withdrawal [4].

### Nocebo effect

The nocebo effect refers to those particular unfavorable health changes that are observed after a nocebo administration or application which are attributed to the nocebo mechanisms exclusively, e.g. expectation, conditioning, observational learning.

### The effect - response controversy

Not all panel members agree on the terminology suggested. One researcher (IK) use the terms “placebo/nocebo response” and “placebo/nocebo effect” in the opposite way [5]. In a recent position paper from the European Academy of Allergy and Clinical Immunology, the term “placebo effect” was defined as “the psychological and physiological benefits of seeking advice and receiving treatment for a medical problem, independently of the prescribed treatment’s pharmacological effects *per se*” [6]*.* Historical and traditional reasons may explain the differences. Here, the vast majority of panelists agreed on the recommended terminology, and, eventually, this is an area that needs to be ironed out in future research.

## Clinical implications

### Should we distinguish the placebo or nocebo response from the placebo or nocebo effect in clinical trials and why?

#### Introduction

Most panelists declare there is a significant difference between the two terms, but not all agree. There is no agreement for the rationale as well. Defenders say that the distinction is important for the same reason that it is important to distinguish between a drug response and a drug effect, with the drug effect as the difference between the drug response and the placebo response. “A drug response is the change that occurs after administration of the drug. The effect of the drug is that portion of the response that is due to the drug’s chemical composition; it is the difference between the drug response and the response to placebo.” [7]. However, in clinical trials both treatment response and treatment effect are collectively used as treatment efficacy reflecting the expected effect size of the treatment in practice. In analyses of clinical trials another metric is used, the “therapeutic gain” representing the difference of the outcome between active and placebo-treated groups [8]. Another metric is the absolute difference between active drug and placebo-treated arms, known as number-needed to treat [9] and the relative efficacy of the treatment, or the relative risk reduction as measures of treatment effect size [10], or both [11]. The terms “treatment response” and “treatment effect” have very limited use in the literature currently, at least in the field of neurology and headache. Therefore, distinguishing placebo response from placebo effect remains controversial for clinical science. On the other hand, all panelists considered it necessary to distinguish placebo/nocebo responses from placebo/nocebo effect in order to investigate underlying potential mechanisms. Thus, the final recommendation is mechanism-based.

#### Recommendation

We recommend distinguishing the two terms (response from effect) because their mechanisms are different. This distinction is crucial for the research (experimental human studies), but may also help practitioners to better interpret the trial results and to modify their practice in order to limit the modifiable nocebo factors listed in appendix.

### What should we call the efficacy and the adverse events recorded in the placebo arms of a clinical trial?

#### Introduction

A systematic review of clinical trials concluded that the median prevalence of AEs in placebo groups was 49.1% (IQR 25.7–64.4%), and the median rate of dropouts due to AEs was 5% (IQR 2.28–8.4%) [12]. There is therefore a question of terminology regarding the AEs recorded in placebo arms. Most panelists agree that the term “response” better describes the favorable and unfavorable outcomes observed after placebo administration in clinical trials.

#### Recommendation

According to the terminology of placebo response, the beneficial outcomes seen in a placebo-treated arm of a clinical trial should be called placebo response. In the same way, any unfavorable outcome seen in the placebo-treated arms of clinical trials should be called nocebo response.

### Environmental stimuli influencing placebo and nocebo responses in clinical trials and practice

The surrounding context that potentially modifies placebo and nocebo responses in clinical science and practice consists of any stimulus or information from the environment (e.g. media, internet, health setting); the treating medical and paramedical personnel or researcher (including the symbols, rituals, and verbal and nonverbal expressions and communications they employ); the patient’s medical condition, or the rationale for the experiment; and the expectations and prior experiences of the patient or volunteer that may vary over time or by education; and the price of a medication or the label on the box, etc. In a clinical trial these factors are, presumably, essentially the same for both the control and verum conditions. And, of course, these same factors are also operative in ordinary clinical practice. In Appendix, a list of these stimuli is presented, divided into modifiable and non-modifiable ones.

## Conclusions

Treatment outcomes are not exclusively attributable to the biological or physical mechanisms of action of the therapeutic interventions *per se*, but additional environmental, random or idiosyncratic variables are also components of the final result. To better understand and investigate this impact a mechanism-based approach is needed. Therefore, to measure the effect size of a treatment that can be attributed exclusively to the mechanism of action of the treatment in question, testing in parallel with an inert treatment is essential. This inert treatment whether it is an agent, a manipulation, a sham stimulation or an inert intervention, should be called *placebo*. To interpret the consequences of placebo administration or application in clinical trials two terms have been in use, the placebo response and the placebo effect. However, these terms have different meanings, refer to different elements and are powered by different mechanisms. This panel of experts, on behalf of the European Headache Federation, aiming to clarify these terms in order to help clinicians and trialists to better interpret the trials’ results, recommends distinguishing the term *response* from *effect*, using the term *placebo response* to describe any favorable outcome observed from before to after placebo administration, and *nocebo response* to describe any unfavorable outcome observed from before to after placebo administration. Several random variables, such as accidental deterioration or improvement in the condition, or accidental involvement with another condition along with idiosyncratic variables that are linked to placebo or nocebo mechanisms are components of the placebo or nocebo responses, while the *placebo* and *nocebo effects* refer to those favorable or unfavorable outcomes observed from before to after a placebo application that are powered by the patient mind entirely, e.g. conditioning, expectations, observational learning, etc.

In human experimental studies, to induce negative outcomes and investigate their underlying mechanisms, inert interventions are administered to participants. These inert agents or interventions that cause unfavorable outcomes although their active ingredients lack this potential, are called *nocebos*. Like placebos, nocebo responses should be distinguished from nocebo effects. In clinical trials there is no direct nocebo application, yet the safety information delivered by the investigators may serve as a nocebo in both arms and may induce AEs that cannot be separated from the drug-related ones. To limit nocebo effects, appropriate modification of the informed consents currently used may be required.

In clinical practice, placebos and nocebos are rarely explicitly administered. However, environmental information, including any stimuli from the entire therapeutic encounter may serve as placebo or nocebo, e.g. the drug or device packaging, color, price, the setting of the medical unit, the patient-doctor relationship and clinician communication style, etc. Physicians should be aware of these variables in order to devise strategies for each individual patient that limit nocebo and increase placebo effects to improve treatment outcomes for the patient.

## Data Availability

There are no original data.

## Appendix

**Table 2:** Environmental factors and stimuli modifying placebo/nocebo effects

Modifiable	Non-modifiable
Patient’s expectations [13]	Previous experiences [14]
Pre-treatment verbal and non-verbal suggestions [15, 16]	Patient’s personality [17]
Speed of treatment titration [18]	Cultural factors [19, 20]
Safety profile of treatment [21]	Age [22]
Patient-doctor relation/communication [23]	Social Media and Internet information [24]
Investigator/physician status [15]	Gender [25]
Affective and cognitive traits [26]	Level of patients’ education [22]
Generic formulations [27, 28]	Genetics [29]
The appearance of drugs or medical devices, e.g. packaging, color, price, drug taste, etc. [30, 31]	
Invasive, non-invasive treatments [32]	
